# SARS-CoV-2 within-host diversity of human hosts and its implications for viral immune evasion

**DOI:** 10.1128/mbio.00679-23

**Published:** 2023-06-05

**Authors:** Binbin Xi, Xi Zeng, Zixi Chen, Jiong Zeng, Lizhen Huang, Hongli Du

**Affiliations:** 1 School of Biology and Biological Engineering, South China University of Technology, Guangzhou, China; University of Calgary, Calgary, Canada

**Keywords:** SARS-CoV-2, within-host diversity, iSNVs, RNA editing, CpG, ZAP, immune evasion

## Abstract

Severe acute respiratory syndrome coronavirus 2 (SARS-CoV-2) is continuously evolving, bringing great challenges to the control of the virus. In the present study, we investigated the characteristics of SARS-CoV-2 within-host diversity of human hosts and its implications for immune evasion using about 2,00,000 high-depth next-generation genome sequencing data of SARS-CoV-2. A total of 44% of the samples showed within-host variations (iSNVs), and the average number of iSNVs in the samples with iSNV was 1.90. C-to-U is the dominant substitution pattern for iSNVs. C-to-U/G-to-A and A-to-G/U-to-C preferentially occur in 5′-CG-3′ and 5′-AU-3′ motifs, respectively. In addition, we found that SARS-CoV-2 within-host variations are under negative selection. About 15.6% iSNVs had an impact on the content of the CpG dinucleotide (CpG) in SARS-CoV-2 genomes. We detected signatures of faster loss of CpG-gaining iSNVs, possibly resulting from zinc-finger antiviral protein-mediated antiviral activities targeting CpG, which could be the major reason for CpG depletion in SARS-CoV-2 consensus genomes. The non-synonymous iSNVs in the *S* gene can largely alter the S protein’s antigenic features, and many of these iSNVs are distributed in the amino-terminal domain (NTD) and receptor-binding domain (RBD). These results suggest that SARS-CoV-2 interacts actively with human hosts and attempts to take different evolutionary strategies to escape human innate and adaptive immunity. These new findings further deepen and widen our understanding of the within-host evolutionary features of SARS-CoV-2.

**IMPORTANCE:**

Severe acute respiratory syndrome coronavirus 2 (SARS-CoV-2), the causative pathogen of the coronavirus disease 2019, has evolved rapidly since it was discovered. Recent studies have pointed out that some mutations in the SARS-CoV-2 S protein could confer SARS-CoV-2 the ability to evade the human adaptive immune system. In addition, it is observed that the content of the CpG dinucleotide in SARS-CoV-2 genome sequences has decreased over time, reflecting the adaptation to the human host. The significance of our research is revealing the characteristics of SARS-CoV-2 within-host diversity of human hosts, identifying the causes of CpG depletion in SARS-CoV-2 consensus genomes, and exploring the potential impacts of non-synonymous within-host variations in the *S* gene on immune escape, which could further deepen and widen our understanding of the evolutionary features of SARS-CoV-2.

## INTRODUCTION

Severe acute respiratory syndrome coronavirus 2 (SARS-CoV-2), the causative pathogen of the coronavirus disease 2019 (COVID-19), is a positive-sense single-stranded RNA virus, possessing a 30-kb genome (1). SARS-CoV-2 is in rapid evolution, and new lineages are continuously emerging. The lineage-defining mutations may increase viral transmissibility, raise viral infectivity, and especially confer SARS-CoV-2 the ability to evade the human immune system, thus bringing great challenges to the control of the virus. It has been demonstrated that the D614G mutation in the S protein, a defining mutation of B.1 lineage (Pango nomenclature), can increase viral transmissibility (2 - 4). In addition, there is compelling evidence that the E484K and N501Y mutations in the S protein can reduce antibody neutralization and render SARS-CoV-2 immune escape capability (5 - 7).

Though it is still unclear how SARS-CoV-2 lineages emerge, the mutations of lineages ultimately emerge inside host cells. Upon entering host cells, SARS-CoV-2 continuously generates new mutations due to different mutation drivers and forms a heterogeneous genomic virus population called quasispecies inside hosts. The newly emerging mutations, called within-host variations (iSNVs), undergo different selective pressures, including those from innate and adaptive immunity (8). Different mutation drivers and selective pressures will leave SARS-CoV-2 with different mutational and genomic signatures. These signatures, for example, the dominant substitution type of iSNVs, the context of different substitution types, the change of CpG content in SARS-CoV-2 genomes, etc., will provide us with important information about the interactions between SARS-CoV-2 and hosts.

It has been reported that SARS-CoV-2 exhibits low within-host diversity (9 - 11), partially due to the nsp14 proofreading activity (12). C-to-U has been demonstrated as the dominant substitution pattern for iSNVs, and the host-directed RNA editing proteins, activation-induced cytidine deaminase/apolipoprotein B mRNA-editing enzyme catalytic polypeptide-like (AID/APOBEC) protein family, mainly APOBEC1, APOBEC3A (A3A), and APOBEC3G (A3G), are speculated to be responsible for the generation of C-to-U substitutions (13 - 18). The substrates’ preference for APOBEC1, A3A, and A3G is 5′-AC-3′, 5′-UC-3′, and 5′-CC-3′, respectively (reviewed in ref [19]). However, there is no clear evidence that C-to-U substitutions occur preferentially in particular motifs.

CpG deficiency, the phenomenon that genomes present low content of CpG dinucleotide, has been documented in many single-stranded RNA viruses, including SARS-CoV-2 (20 - 23). In mammals, zinc-finger antiviral protein (ZAP), a key component of innate antiviral response, binds specifically to CpG dinucleotides in viral RNA genomes and recruits its cofactors, KHNYN and TRIM25, to degrade the viral RNA, thus inhibiting virus replication (24 - 26). Additionally, ZAP is likely the primary cellular protein that targets CpG dinucleotides in viral RNA genomes (22). Therefore, CpG deficiency is likely a way to evade ZAP-mediated cellular antiviral defense. A global analysis of protein–RNA interaction in SARS-CoV-2-infected cells shows that ZAP is significantly upregulated, indicating the ZAP protein plays a vital role in the antiviral response against SARS-CoV-2 (27). SARS-CoV-2 must adapt to the human immune environment following the transmission from intermediate or reservoir hosts to humans. Hence, although SARS-CoV-2 has exhibited extreme genomic CpG deficiency, there is still modest CpG depletion in its genomes as evolving (8, 23). In other words, the content of CpG dinucleotides in SARS-CoV-2 genomes has decreased since it was discovered. However, how CpG depletion occurs remains unclear.

Based on our previous research on the evolution of SARS-CoV-2 consensus genomes (28 - 31), we investigated the characteristics of SARS-CoV-2 within-host diversity and the implications of iSNVs for viral immune evasion using almost 2,00,000 raw genome sequencing data of SARS-CoV-2. Our work provided insights into the possible mutation drivers or their mutational signatures for iSNVs and the evolutionary strategies of SARS-CoV-2 to escape human innate and adaptive immunity. These findings would be beneficial for understanding viral evolution and immune escape.

## MATERIALS AND METHODS

### Data set

Raw SARS-CoV-2 genome sequencing data were retrieved from the European Nucleotide Archive (https://www.ebi.ac.uk/ena) Project PRJEB37886, a SARS-CoV-2 sequencing data sharing project initiated by the COVID-19 Genomics UK Consortium. The metadata of PRJEB37886, containing 2,523,549 SARS-CoV-2 genome sequencing records, was downloaded on 22 May 2022. Sequencing data that were obtained by amplicon library strategy and sequenced on the Illumina NovaSeq 6,000 instrument, and with sequencing depth bigger than 10,000× were retained, resulting in 1,508,953 records. A total of 2,00,000 records were randomly sampled from the remaining records by the function numpy.random.choice of the Python Numpy package v1.19.5, and the BAM format files of the sampled records were downloaded, constituting the sequencing dataset of the study.

### Variant calling, mutation filtration, and mutation annotation

The ARTIC primers were trimmed by iVar v1.3.1 (32) with default settings. Mutations were detected by Freebayes v1.3.6 (33) (parameters: -p 1 -q 20-m 20 -V -F 0.005 -C 5 -min-coverage 10). NC_045512.2 (NCBI, https://www.ncbi.nlm.nih.gov/) was used as the reference sequence. Mutations meeting one of the following criteria were removed: (i) positions not in the range of 55–29,826 (because of the amplification restriction of ARTIC); (ii) insertions and deletions; (iii) mutation positions with <100× sequencing depth; (iv) mutations with less than five forward or reverse supporting reads; (v) mutations with alternate allele frequency (AAF) less than 0.05; and (vi) 67 highly shared positions (Table S1). Besides, if there were more than one mutation at a genome position in a sample, the mutation with the highest AAF would be retained. The cleaned mutations were divided into two groups according to their AAF: SNVs with AAF higher than 0.95, which were defined as the mutations of the viral consensus genomes before infecting hosts, and iSNVs with AAF lower than 0.95, which were defined as the mutations that were newly generated within infected individuals. Mutations were annotated using ANNOVAR (34).

### The probability of mutation times at a particular genome position in the dataset

The substitution rate of SARS-CoV-2 is about 1.1 × 10^−3^ substitutions per site per year (35). The average incubation period (the interval between infection and symptom onset) of SARS-CoV-2 is about 5.2 d (36). Therefore, the substitution rate of SARS-CoV-2 is about 1.567 × 10^−5^ substitutions per site per incubation period. Mutation times of a particular genome position in the dataset could be modeled as a Poisson distribution with λ equal to 3.067. The probability that a position mutated *k* times was calculated as follows:


$$P\left(X=k\right)=\frac{\lambda^{k}}{k!}e^{-\lambda },\;k=0,1,...14$$

where *k* is the mutation times at a particular position; *P (X = k*) is the probability that the position mutates *k* times in the dataset.

### iSNVs density

The density of iSNVs across the genome was counted using a sliding window of 100 bp and moved forward by 1 bp each time. The density of iSNVs of a gene was calculated as follows:


$$density=\frac{N_{G}}{195,739*L_{G}}*100$$

where $N_{G}$ is the number of iSNVs in the gene, 195,739 is the total number of samples of the dataset, and $L_{G}$ is the length of the gene.

### Normalized proportion

The count of each substitution type (e.g., the C-to-U substitution) was normalized by the corresponding nucleotide count (e.g., the C nucleotide) in the reference genome (NC_045512.2, positions from 55 to 29,826). For the −1 and +1 positions’ nucleotide contexts of the 12 substitution types, the mutation formed a dinucleotide with its −1 (or +1) position nucleotide. We counted the number of different dinucleotides for the 12 substitution types. The count of each dinucleotide was then normalized by the occurrence of the corresponding dinucleotide count in the reference genome. The normalized proportion was calculated according to their normalized frequency.

### Calculation of Ka/Ks

The number of synonymous and non-synonymous substitutions was estimated for the coding regions and all genes of the SARS-CoV-2 reference sequence (37). The ratio of non-synonymous to synonymous (Ka/Ks) for iSNVs in the coding region and different genes of one quasispecies were calculated according to the method of ref (9). Briefly, the Ka/Ks of a genomic region *G* was calculated as follows (9):


$$\frac{\sum_{p\in G}i_{p}^{N}}{T_{G}^{N}}/\frac{\sum_{p\in G}i_{p}^{s}}{T_{G}^{S}}$$

where $i_{p}^{N}$ is the fraction (AAF) of iSNVs at *P* that is non-synonymous or 0 if there are no iSNVs at *P*; $T_{G}^{N}$ is the total number of potential non-synonymous substitutions in *G*; and the denominator replaces N with *S* to represent synonymous substitutions (9).

### Prediction of viral escape capability

Samples with at least one non-synonymous iSNV in the *S* gene were included, resulting in 11,038 samples. The SNVs with AAF higher than 0.95 in the *S* gene of a sample constituted the mutational background. The background amino acid sequence of the S protein of each sample was obtained according to their non-synonymous SNVs in the *S* gene. Then the corresponding single-point mutation amino acid sequence was generated for each non-synonymous iSNV in the *S* gene based on the sample’s background amino acid sequence. The viral escape capability of these S protein amino acid sequences, including the background sequences and the corresponding single-point mutation sequences, was predicted by viral escape (38) with default settings and using the wild-type S protein as a reference.

The 11,038 samples were clustered according to their background amino acid sequence. The prediction results for the top eight clusters (Table S2) with the largest sample size were visualized in Fig. 5.

## RESULTS

### SARS-CoV-2 is with low within-host diversity, and iSNVs density along the genome is variable

**Fig 1:**
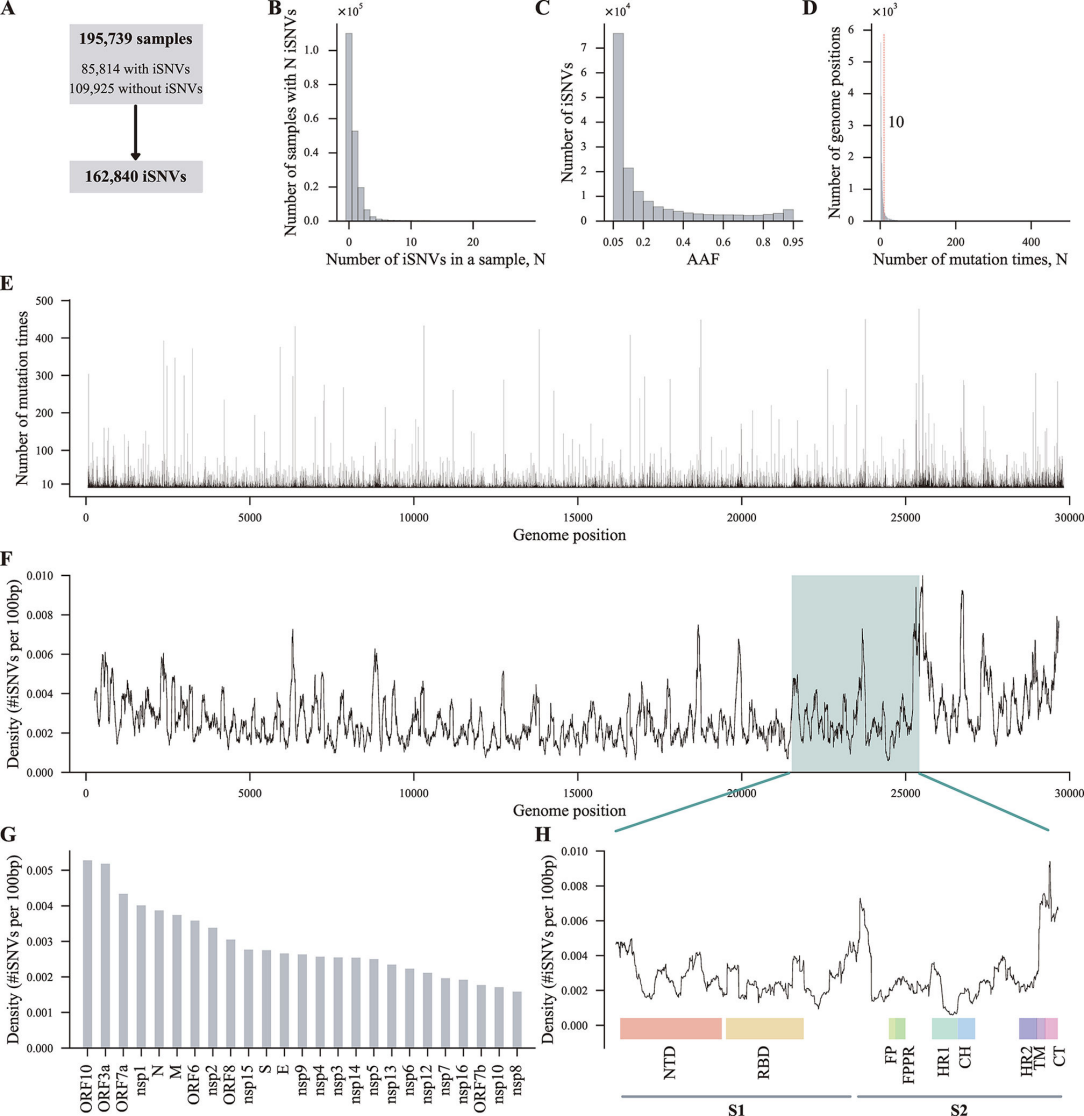
SARS-CoV-2 within-host diversity and iSNVs density along the genome. (**A**) Overview of the iSNVs dataset. (**B**) The number of samples that had *N* iSNVs. (**C**) The distribution of AAFs of all iSNVs. The histogram represents the number of iSNVs of different AAF ranges. (**D**) The number of genome positions that mutated *N* times. We modeled the probability distribution of the number of mutation times at a particular genome position based on the assumption that mutations occur randomly across the SARS-CoV-2 genome (Fig. S1). The vertical line in red represents the theoretically largest number of mutation times at a particular genome position in the dataset. (**E**) The number of mutation times at different genome positions. (**F**) iSNVs density along the SARS-CoV-2 genome, counted using a sliding window of 100 bp and moving forward by 1 bp each time. The dark cyan shade represents the genome region of the *S* gene. 5′ UTR and 3′ UTR were excluded. (**G**) The iSNVs densities of different genes. Genes encoding non-structural proteins: *nsp1–nsp10* and *nsp12–nsp16*; genes encoding accessory proteins: *ORF3a*, *ORF6*, *ORF7a*, *ORF7b*, *ORF8*, and *ORF10*; genes encoding structural proteins: *S*, *M*, *N*, and *E*. (**H**) The iSNVs density of the *S* gene, a magnified view of the dark cyan region of (**F**). UTR, (untranslated region); S1, S1 subunit; S2, S2 subunit; FP, fusion peptide; FPPR, fusion peptide-proximal region; HR1, heptad repeat 1; CH, central helix; HR2, heptad repeat 2; TM, transmembrane anchor; CT, cytoplasmic tail.

We obtained a convincing SARS-CoV-2 iSNVs dataset using several stringent variant filtration criteria. Overall, there were 195,739 samples in the dataset (Fig. 1A), of which 109,925 samples had no iSNVs, and 85,814 samples (~44%, 85,814/195,739) exhibited at least 1 iSNVs (median 1, mean 1.90, and interquartile range 1.0–2.0, Fig. 1A and B), totally 162,840 iSNVs. The AAFs of the iSNVs were mainly distributed between 0.05 and 0.10 (Fig. 1C). In addition, the number of iSNVs gradually decreased as AAF increased and was relatively stable when AAF was larger than 0.4 and smaller than 0.9 but slightly increased when AAF was larger than 0.90 (Fig. 1C). These results indicated that SARS-CoV-2 had low within-host diversity, and most iSNVs did not make their subpopulations dominant inside hosts. Of course, these results were affected by the AAF threshold, 0.05 in the present study, and the time interval between sampling and infection to some extent.

Recurrent mutation refers to the same mutation that independently occurs in different samples. Here, we defined recurrent mutation as mutations independently occurring at the same genome position in different samples regardless of their exact mutation types. Supposing iSNVs occur randomly across the genome, the number of mutation times at a particular genome position should not exceed about 10 in the dataset (Fig. S1, see Materials and Methods). We counted the number of mutation times at each genome position and found most genome positions mutated no more than 10 times (Fig. 1D; Fig. S1). Nonetheless, there were also positions mutated exceeding 10 times, and these positions were distributed along the entire viral genome (Fig. 1D and E). This observation suggested that there might be some mutational drivers that caused mutations at these positions.

To understand the distribution of the iSNVs across the genome, we calculated the iSNVs density along the genome. Strikingly, SARS-CoV-2 iSNVs were distributed unevenly throughout the genome (Fig. 1F and G). The genes of SARS-CoV-2 are divided into three categories, non-structural genes (*nsp1–nsp10* and *nsp12–nsp16*), accessory genes (*ORF3a*, *ORF6*, *ORF7a*, *ORF7b*, *ORF8*, and *ORF10*), and structural genes (*S*, *M*, *N*, and *E*), according to their functions. Most genes encoding the structural and accessory proteins at the 3′ end of the genome showed high variability (Fig. 1F and G). In contrast, most genes at the 5′ end of the genome, encoding nonstructural proteins, displayed lower variability (Fig. 1F and G). *ORF10* and *ORF3a*, encoding two accessory proteins, exhibited the highest iSNVs densities (0.0053 and 0.0052 iSNVs/100 bp, respectively), about 3.3 times that of *nsp8* (0.0016 iSNVs/100 bp) (Fig. 1G). Of note, the junction regions of the S1 and S2 subunits and the CT domain of the S2 subunit showed relatively high densities of iSNVs (Fig. 1H).

### C-to-U/G-to-A and A-to-G/U-to-C frequently occur in 5*'*-CG-3*'* and 5*'*-AU-3*'* motifs, respectively

**Fig 2:**
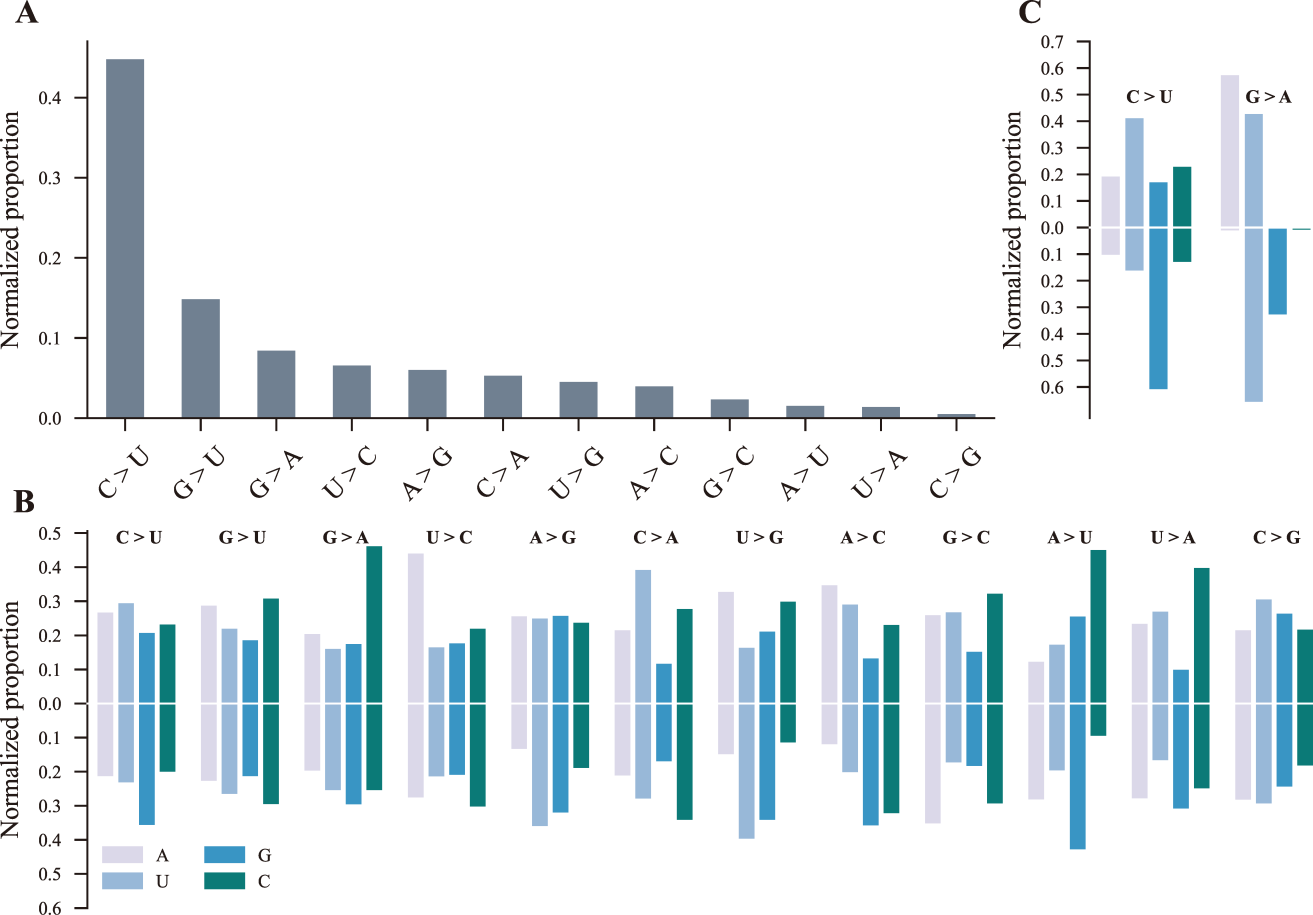
Substitution spectra of iSNVs and the contexts of different substitution types. (**A**) The normalized proportion of the 12 substitution types of the iSNVs. The iSNVs were divided into 12 different substitution types according to their reference and alternate nucleotides. The number of iSNVs for each substitution type was counted and normalized by the number of the corresponding reference nucleotide in the reference genome (NC_045512.2, positions from 55 to 29,826). (**B**) Sequence contexts of the 12 substitution types of all iSNVs at the −1 position (the upper panel) and the +1 position (the lower panel). (**C**) Sequence contexts of the C-to-U and G-to-A substitution types of recurrent mutations with mutation times ≥100 at the −1 position (the upper panel) and the +1 position (the lower panel). The caption of (**C**) was as same as that of (**B**).

Here, we characterized the features of the substitution patterns of SARS-CoV-2 iSNVs. To eliminate the influence of the skewed nucleotide composition of the SARS-CoV-2 genome, we calculated the normalized proportion of each substitution type (see Materials and Methods). Consistent with the previous study (14), C-to-U and G-to-U were the two dominant substitution types of iSNVs (Fig. 2A). Additionally, we observed a similar normalized proportion of G-to-A, U-to-C, A-to-G, C-to-A, U-to-G, and A-to-C substitutions (Fig. 2A).

Sequence contexts of substitution types can provide clues about their corresponding mutation drivers. Therefore, we explored the −1 and +1 positions’ contexts of the 12 substitution types (Fig. 2B). C-to-U and G-to-A substitutions preferentially occurred in 5′-CG-3′ motifs (Fig. 2B). G-to-U exhibited no marked context preference at both the −1 and +1 positions, while C-to-A substitutions occurred in 5′-[U]C[C]-3′ motifs more frequently (Fig. 2B). U-to-C and A-to-G substitutions more likely occurred in 5′-AU-3′ motifs (Fig. 2B). In addition to U, A-to-G substitutions displayed a preferential context for G at the +1 position (Fig. 2B). Furthermore, U-to-G substitutions occurred more frequently in 5′-[A]U[U/G]-3′ motifs, while A-to-C substitutions preferentially occurred in 5′-[A]A[G/C]-3′ motifs (Fig. 2B). It has been proposed that C-to-U and G-to-A (C-to-U occurring on the minus strand) are the results of APOBECs-mediated deamination (13, 14). We thus explored the sequence contexts of C-to-U and G-to-A substitutions of recurrent mutations with mutation times ≥100 (Table S3). Except for the obvious G-skewed context at the +1 position resembling that of all iSNVs, C-to-U exhibited a marked preference for U at the −1 position (Fig. 2C). However, the contexts at the −1 and +1 positions of G-to-A substitutions of recurrent mutations were largely different from that of G-to-A of all iSNVs. The G-to-A −1 position displayed G and C depletion, and the +1 position displayed A and C depletion. In addition, G-to-A of recurrent mutations appeared more likely to occur in 5′-[A/U]G[U/G]-3′ motifs (Fig. 2C). These differences suggested that more than one mutational driver was likely responsible for G-to-A substitutions.

### iSNVs of SARS-CoV-2 are under negative selection

**Fig 3:**
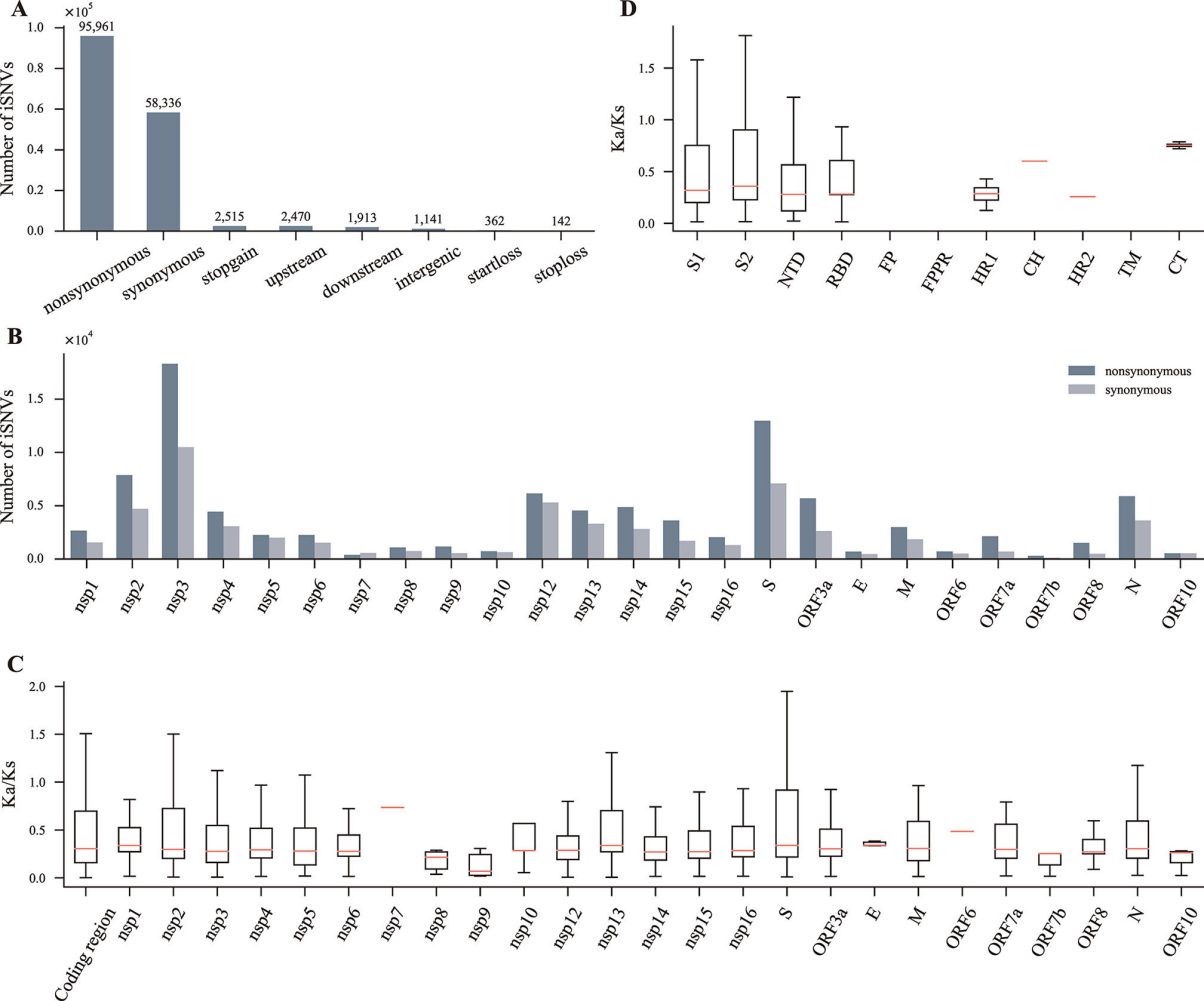
Selection on SARS-CoV-2 iSNVs. (**A**) The number of iSNVs of different mutation types. nonsynonymous, iSNVs causing changes in the encoding amino acids; synonymous, iSNVs causing no change in the encoding amino acids; stopgain, iSNVs causing the codon to become a stop codon; upstream, iSNVs occurring in the 5′ UTR; downstream, iSNVs occurring in the 3′ UTR; intergenic, iSNVs occurring in regions between two genes; startloss, iSNVs causing the loss of the initiation codon; stoploss, iSNVs causing the loss of stop codons. (**B**) The number of non-synonymous and synonymous iSNVs of different genes. (**C**) The Ka/Ks of the coding region and different genes of SARS-CoV-2. (**D**) The Ka/Ks of the S1 subunit, S2 subunit, and different domains of the S protein. No available Ka/Ks data for FP, FPPR, and TM. The boxes represent the median (the line in salmon) and the first quartile (**Q1**) to the third quartile (**Q3**), with whiskers extending from the box by 1.5× interquartile range. FP, fusion peptide; FPPR, fusion peptide-proximal region; HR1, heptad repeat 1; CH, central helix; HR2, heptad repeat 2; TM, transmembrane anchor; CT, cytoplasmic tail.

Since various iSNVs densities along the genome of SARS-CoV-2 were detected, we further investigated the selection acting on SARS-CoV-2 iSNVs. Of the 162,840 iSNVs, most were non-synonymous or synonymous, and the number of non-synonymous iSNVs was about 1.6 times that of synonymous iSNVs (Fig. 3A). The number of non-synonymous iSNVs of each gene was also more than that of synonymous iSNVs (Fig. 3B). We calculated the ratio of non-synonymous to synonymous substitutions (Ka/Ks) for each gene. Consistent with the previous study (9), the Ka/Ks of the coding region and genes were all lower than 1, indicating they were under negative (purifying) selection (Fig. 3C). In addition, we calculated the Ka/Ks of different subunits and domains of the S protein and got similar results (Fig. 3D). Of note, the Ka/Ks of most *S* genes was less than 1.0, indicating the *S* genes of SARS-CoV-2 in most individuals were under negative selection. In addition, the distribution of the Ka/Ks showed a large range of variations, suggesting great differences in the strength of selection on the iSNVs in the *S* gene in different individuals (Fig. 3C).

### Faster loss of CpG-gaining iSNVs is responsible for CpG depletion in SARS-CoV-2 consensus genomes

**Fig 4:**
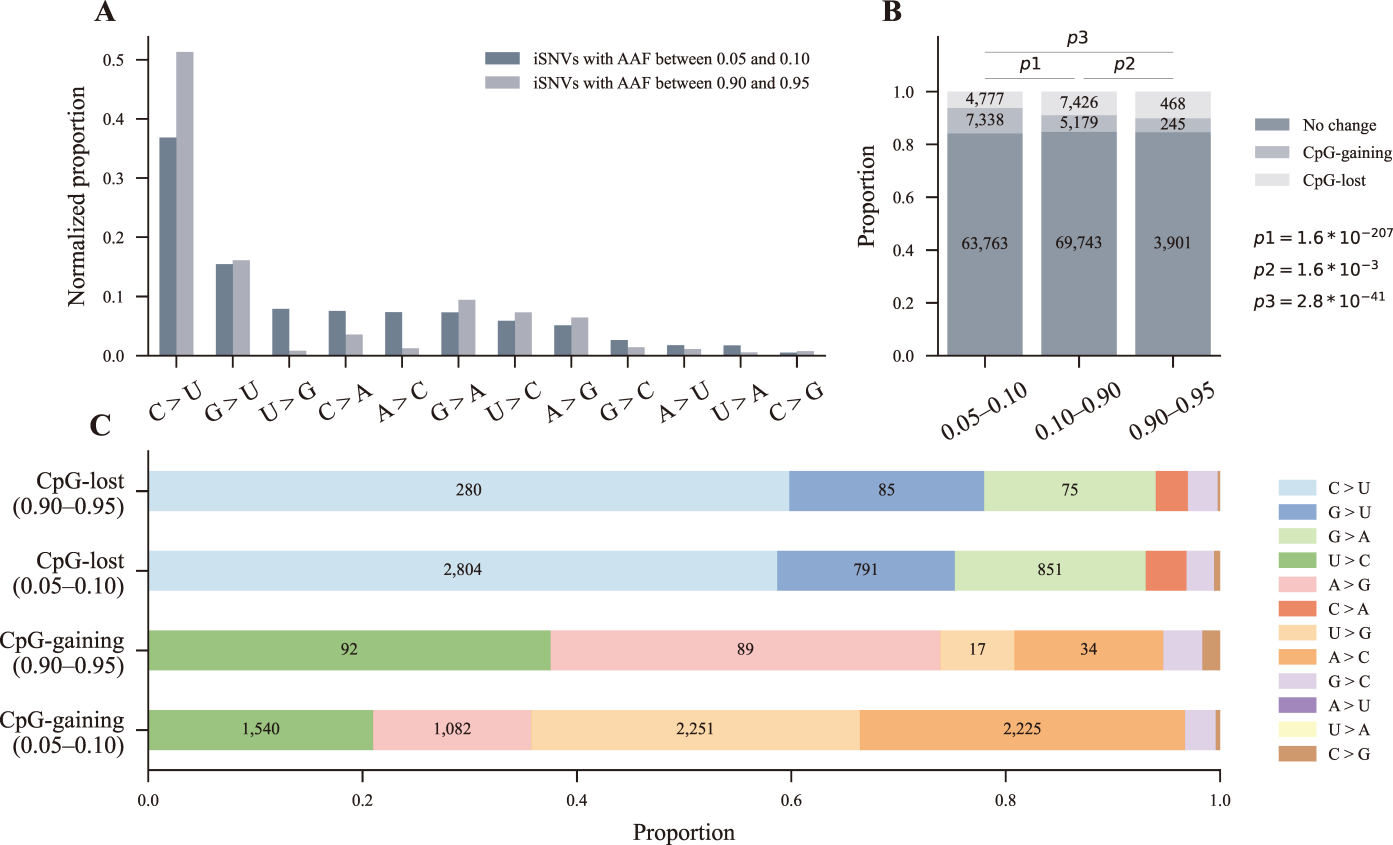
Impacts of iSNVs on CpG content of genome sequences. (**A**) The normalized proportion of the 12 substitution types of the random iSNVs group (iSNVs with AAF between 0.05 and 0.10) and the advantageous iSNVs group (iSNVs with AAF between 0.90 and 0.95). (**B**) The number of CpG-gaining, CpG-lost, and no change iSNVs of the three iSNVs groups (iSNVs with 0.05–0.10, 0.10–0.90, and 0.90–0.95 AAF). iSNVs were divided into three categories, no change iSNVs, CpG-gaining iSNVs, and CpG-lost iSNVs, according to their impacts on the CpG content of genome sequences (see below). Significant differences in the proportion of the three categories of iSNVs between any two groups (p1 = 1.6 × 10^−207^, p2 = 1.6 × 10^−3^, p3 = 2.8 × 10^−41^, and 2 × 3 contingency table). No change iSNV, the iSNV resulting in neither the gaining of a CpG nor the loss of a CpG; CpG-gaining iSNV, the iSNV resulting in the gaining of a CpG; CpG-lost iSNV, the iSNV resulting in the loss of a CpG; (**C**) The contribution of different substitution types to the gaining and loss of CpGs for the random iSNVs group (iSNVs with AAF between 0.05 and 0.10) and the advantageous iSNVs group (iSNVs with AAF between 0.90 and 0.95).

One tenet of evolution is that mutations occur randomly. Some mutations are advantageous, which can increase viral fitness, while some are deleterious, decreasing viral fitness (39). Consequently, the AAFs of advantageous mutations shift toward higher frequencies, while those of deleterious mutations shift toward lower frequencies. In addition, some advantageous mutations may have low AAFs because of the short interval between infection and sampling. Thus, we hypothesized that iSNVs with AAF higher than 0.90 were more advantageous and had experienced selection, while iSNVs with AAF lower than 0.10 were more random and were undergoing selection. Here, iSNVs with AAF between 0.90 and 0.95 were called advantageous iSNVs, and iSNVs with AAF between 0.05 and 0.10 were called random iSNVs. Strikingly, there were obvious differences in the composition of substitution types between the groups of advantageous iSNVs and random iSNVs (Fig. 4A). Especially, U-to-G and A-to-C substitutions almost disappeared in the group of advantageous iSNVs (Fig. 4A).

To investigate the effects of iSNVs on CpG content in genomes, we divided iSNVs into three categories: CpG-gaining iSNVs, CpG-lost iSNVs, and iSNVs causing no change in CpG content (Fig. 4B). We then counted the number of iSNVs in each category for the groups of advantageous iSNVs and random iSNVs individually. Overall, about 15.6% (25,433/162,840) iSNVs impacted CpG content in SARS-CoV-2 genomes (Fig. 4B). There were remarkable differences in the proportion of the three iSNV categories between the advantageous iSNVs group and the random iSNVs group (Fig. 4B, *P* = 2.8 × 10^−41^ and 2 × 3 contingency table). The advantageous iSNVs group showed a higher proportion of CpG-lost iSNVs (10.1%) and a lower proportion of CpG-gaining iSNVs (5.3%) than that of the random iSNVs group (6.3% for CpG-lost iSNVs and 9.7% for CpG-gaining iSNVs) (Fig. 4B; Table S4). Additionally, the proportion of CpG-lost iSNVs in the advantageous iSNVs was 1.6 times that of the random iSNVs, while the proportion of CpG-gaining iSNVs in the advantageous iSNVs was only 0.54 times that of the random iSNVs (Fig. 4B; Table S4). Combining the above observation that SARS-CoV-2 iSNVs were under negative selection, these results indicated CpG-gaining iSNVs experienced much stronger negative selection and were lost faster.

We then sought to determine the major substitution types contributing to the gaining and the loss of CpGs, respectively. As expected, C-to-U, G-to-U, and G-to-A, the three most abundant substitution types, contributed the most to the loss of CpGs (Fig. 2A; Fig. 4C). In addition, the contribution of the above three substitution types to the loss of CpGs displayed little difference between the random and advantageous iSNVs groups (Fig. 4C). As for gaining CpGs, U-to-C, A-to-G, U-to-G, and A-to-C provided the most contribution (Fig. 4C). However, the contribution of the same substitution type to gaining CpGs in the two groups of iSNVs was largely different (Fig. 4C). In the random iSNVs, U-to-G and A-to-C were the two primary substitution types contributing to the gaining of CpGs. In comparison, U-to-C and A-to-G became the two main substitution types, and U-to-G and A-to-C became much less contributing to the gaining of CpGs in the advantageous iSNVs (Fig. 4C), suggesting U-to-G and A-to-C substitutions in the group of advantageous iSNV have likely experienced stronger negative selection than U-to-C and A-to-G of CpG-gaining iSNVs.

### Non-synonymous iSNVs in the *S* gene confer SARS-CoV-2 the potential ability to escape human adaptive immunity

Since the S protein is the major target of the human adaptive immune system (40, 41), the non-synonymous iSNVs in the *S* gene are likely to increase the SARS-CoV-2 capability of immune escape. Therefore, we estimated the effects of the non-synonymous iSNVs in the *S* gene on the capability of viral immune escape. Overall, 11,038 samples exhibited at least one non-synonymous iSNVs in the *S* gene. For each non-synonymous iSNV, we first generated a single-point mutation amino acid sequence based on its background amino acid sequence (see Materials and Methods). We then estimated the antigenic and fitness changes of each background amino acid sequence and single-point mutation amino acid sequence compared with the wild-type S protein sequence. iSNVs were clustered according to their mutational background, and iSNVs in the same cluster had the same mutational background. The predicted viral escape ability of each iSNV was compared with that of its mutational background to estimate whether the iSNV could increase the ability of immune escape.

**Fig 5:**
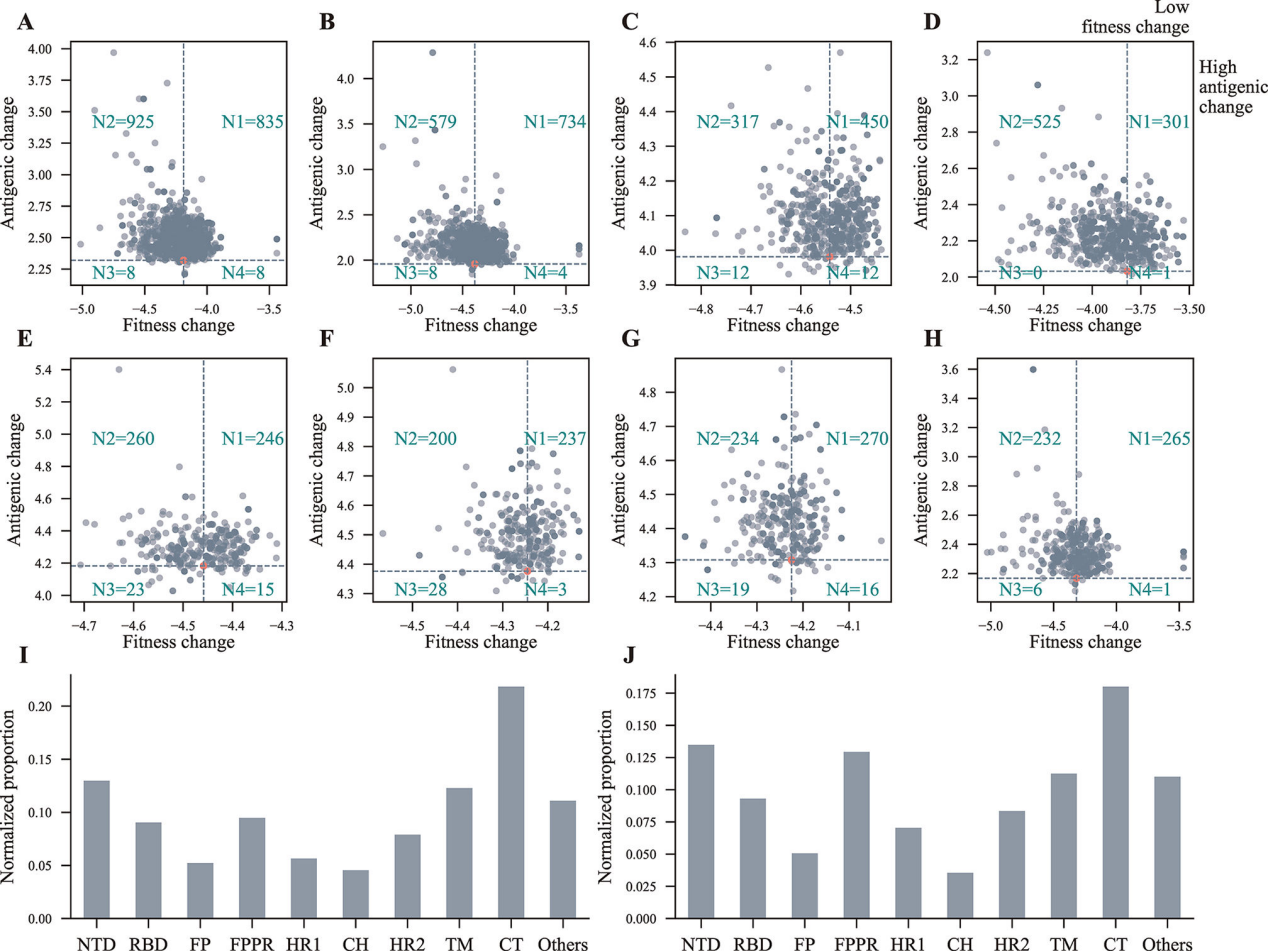
Prediction of the immune escape ability of non-synonymous iSNVs in the *S* gene. (**A–H**) The predicted immune escape ability of the non-synonymous iSNVs of the eight major clusters. All non-synonymous iSNVs in the *S* gene were clustered according to their mutational backgrounds, and iSNVs in the same cluster had the same mutational background. iSNVs of each cluster were divided into four groups by comparing their antigenic change and fitness change to that of their mutational background (see below). Here, only the results for the top eight clusters were visualized. The dot in salmon represents the predicted result of the background amino acid sequence of the S protein. The mutational backgrounds of the eight clusters are listed in Table S2. N1 group, iSNVs with fitness change lower than and antigenic change greater than that of their mutational background; N2 group, iSNVs with fitness change greater than and antigenic change greater than that of their mutational background; N3 group, iSNVs with fitness change greater than and antigenic change lower than that of their mutational background; N4 group, iSNVs with fitness change lower than and antigenic change lower than that of their mutational background. (**I**) The enrichment spectra of iSNVs belonging to the N1 and N2 groups of all clusters (not just the above eight clusters) at different domains of the S protein. (**J**) The enrichment spectra of iSNVs belonging to the N1 group of all clusters (not just the above eight clusters) at different domains of the S protein. iSNVs belonging to the N1 group of all clusters and enriched in the NTD and RBD domains were listed in Table S5. The number of iSNVs of each domain was normalized by the nucleic acid length of the corresponding domain.

Strikingly, most non-synonymous iSNVs in the *S* gene (the N1 and N2 groups) caused greater antigenic change than their mutational backgrounds (Fig. 5A through H). If both the fitness change and antigenic change were considered, there were still lots of iSNVs belonging to the N1 group, in which iSNVs had lower fitness and greater antigenic changes than their mutational backgrounds (Fig. 5A through H). Furthermore, many iSNVs belonging to the N1 and N2 groups were distributed in the amino-terminal domain (NTD) and receptor binding domain (RBD) of the S1 subunit (Fig. 5I). If only considering iSNVs belonging to the N1 group, we also observed a similar distribution (Fig. 5J). Additionally, more iSNVs were distributed in the N1 group of the advantageous iSNVs compared with the random iSNVs group (Fig. S2). Taken together, lots of non-synonymous iSNVs in the *S* gene could cause changes in the antigen epitopes of the RBD and NTD of the S protein, enhancing SARS-CoV-2’s ability to evade the attack from neutralizing antibodies and thus conferring SARS-CoV-2 the ability to escape adaptive immunity.

## DISCUSSION

In the present study, by using about 2,00,000 SARS-CoV-2 raw genome sequencing data, we investigated the characteristics of SARS-CoV-2 within-host diversity, the mutational signatures for different substitution types of iSNVs, the causes of CpG depletion in SARS-CoV-2 consensus genomes, the selection against iSNVs, and the effects of the *S* gene non-synonymous iSNVs on viral immune escape.

Consistent with previous studies (9 - 11), SARS-CoV-2 displayed low within-host diversity, with ~56% of samples exhibiting no iSNV and only 1.90 iSNVs for samples having iSNVs on average, and most iSNVs were with low AAFs (Fig. 1A through C). Several factors likely influence the SARS-CoV-2 within-host diversity. First, SARS-CoV-2 has a short incubation period (36). If samples are taken as soon as symptom onset, the virus replicates only for a short time inside hosts, and there will be a small number of iSNVs. Second, the nsp14 protein can proofread and remove mismatched nucleotides and decrease the number of iSNVs (12). Third, iSNVs of most quasispecies are under negative selection (Fig. 3C), which means many newly generated iSNVs are quickly eliminated, and thus the genetic diversity of SARS-CoV-2 quasispecies is reduced. And fourth, in the present study, we only considered iSNVs with AAF larger than 0.05, but there also existed iSNVs with AAF less than 0.05. Hence, the SARS-CoV-2 within-host diversity is underestimated to some extent.

In mammals, RNA editing refers specifically to the deamination of cytosine to uracil (C-to-U) and adenosine to inosine (A-to-I, where I can be thought of as G), and it plays vital roles in viral restriction (19). A-to-I editing is mediated by the adenosine deaminase acting on RNA (ADAR) protein family, which selectively targets dsRNA, while C-to-U editing is mainly performed by the APOBEC protein family, which selectively targets ssRNA (19, 42). SARS-CoV-2 C-to-U and G-to-A substitutions (possible C-to-U substitutions occurring on the minus strand) have been proposed as the results of APOBEC1-mediated or APOBEC3-mediated RNA editing (13). In addition, C-to-U substitutions of iSNVs were reported to preferentially occur downstream from adenosines (As) or uridines (Us), resembling signatures of APOBEC1-mediated or APOBEC3-mediated deamination (13, 19). However, we only observed slight A or U enrichment at the C-to-U −1 position (Fig. 2B). By contrast, we observed remarkable G enrichment at the C-to-U +1 position (Fig. 2B). Thus, C-to-U substitutions occur more frequently within 5′-CG-3′ motifs rather than 5′-[A/U]C-3′ motifs (13). If C-to-U substitutions mainly result from APOBECs-mediated RNA editing (13), 5′-CG-3′ is a more representative signature for APOBECs-mediated SARS-CoV-2 RNA editing. Otherwise, this signature likely suggests that other potential mutational drivers responsible for C-to-U substitutions have a CpG dinucleotide preference. It has also been proposed that ADARs are responsible for the A-to-G and U-to-C substitutions (possible A-to-G substitutions occurring on the minus strand) (13). We observed that A-to-G/U-to-C substitutions frequently occurred in 5′-AU-3′ motifs (Fig. 2B). If ADARs are the primary mutational driver for A-to-G substitutions (13), ADARs likely prefer to edit adenosines (As) within 5′-AU-3′ motifs.

ZAP is an important member of innate immunity, and it inhibits viral replication by specifically binding to CpG dinucleotide and mediating the degradation of the viral RNA genome (24, 25). It has been reported that SARS-CoV-2 displays extreme genomic CpG deficiency (23). Nonetheless, SARS-CoV-2 still exhibits modest CpG depletion in genomes, likely due to adaptive evolution to the human immune environment (8). The present study found that ~15.6% iSNVs affected the CpG dinucleotide content in SARS-CoV-2 genomes (Fig. 4B; Table S4). We detected signatures of faster loss of CpG-gaining iSNVs, possibly resulting from ZAP-mediated antiviral activities targeting CpG (Fig. 4B). In addition, current studies have demonstrated that the transmission bottleneck of SARS-CoV-2 is narrow (9, 10), meaning the newly generated iSNVs are hard to be transmitted, and most often, only the iSNVs with high AAFs will be transmitted (9). Considering that there is a much smaller proportion of CpG-gaining iSNVs in the group of advantageous iSNVs, CpG-gaining iSNVs should be with less chance to be transmitted and be fixed in viral populations than CpG-lost iSNVs, which likely result in the CpG depletion in SARS-CoV-2 consensus genomes. It has been observed that, after a long evolution history within the human host, the frequency of CpG dinucleotides in the influenza viruses has been strongly reduced to adapt to its host (21). Similarly, the modest CpG depletion in SARS-CoV-2 genomes reflects the adaptation to the human host (8). This adaptation, in turn, is likely beneficial for SARS-CoV-2 to invade the ZAP-mediated antiviral response to some extent.

The S protein of SARS-CoV-2 is the principal target of natural infection-induced or vaccine-induced neutralizing antibodies (40, 41). As evolving, many SARS-CoV-2 lineages have emerged and accumulated many mutations in the S protein, some of which can largely alter the epitopes of the S protein and reduce the antibody neutralization potency against SARS-CoV-2 (40). Therefore, a dramatic feature of SARS-CoV-2 is its increasing immune escape ability as evolving (43, 44). Most genes of SAR-CoV-2 are under negative selection, and the *S* gene is not an exception (Fig. 3C). Under negative selection, deleterious iSNVs should be quickly eliminated. Therefore, the remaining non-synonymous iSNVs in the *S* gene should likely increase the immune escape ability of the virus. Indeed, we observed that lots of non-synonymous iSNVs in the *S* gene have the potential to cause greater antigenic change without reducing viral fitness compared with their mutational backgrounds (Fig. 5A through H; Table S2).

Furthermore, many of these iSNVs were distributed in the NTD and RBD domains of the S1 subunit, two major targets for neutralizing antibodies (40, 41) (Fig. 5J). These indicate that the non-synonymous iSNVs in the *S* gene constitute a mutational pool of immune escape. Once some of these iSNVs are transmitted and fixed in the virus population, the immune escape ability of SARS-CoV-2 can be further enhanced. Therefore, surveillance of within-host variations is also needed in addition to monitoring genome-wide mutations.

In summary, though the within-host diversity of SARS-CoV-2 is low, it has huge implications for viral immune evasion. By studying SARS-CoV-2 within-host diversity of human hosts, we find that SARS-CoV-2 takes different evolutionary strategies to escape innate and adaptive immunity. These new findings provide comprehensive insights into SARS-CoV-2 within-host evolutionary characteristics. In the present study, we focused primarily on the implications of iSNVs on viral immune escape. The effects of iSNVs on other phenotypes of SARS-CoV-2 need further study.

## Data Availability

All sequencing data were retrieved from a public database (ENA, project PRJEB37886). All other data are available in the main text and supplemental material.

## SUPPLEMENTAL MATERIAL

Figure S1 mbio.00679-23-s0001.pdf The probability distribution of mutation times at a particular genome position. Mutation times of a particular genome position in the dataset were modeled as a Poisson distribution with λ equal to 3.067, assuming that mutations occur randomly across the SARS-CoV-2 genome.

Figure S2 mbio.00679-23-s0002.pdf Prediction of immune escape ability of the random and advantageous iSNVs in the *S* gene. (A–D) The predicted results of the random iSNVs (iSNVs with AAF between 0.05 and 0.10). (E–H) The predicted results of the advantageous iSNVs (iSNVs with AAF between 0.90 and 0.95). (A) and (E), (B) and (F), (C) and (G), and (D) and (H) had the same mutational background, respectively. Furthermore, (A), (B), (C), and (D) had the same mutational backgrounds as Figure 5A, B, C, and D, respectively. The remaining four clusters in Figure 5 (Figure 5E through H) were not considered because of too few iSNVs with AAF between 0.90 and 0.95 in these clusters.

Table S1 mbio.00679-23-s0003.xlsx The highly shared positions.

Table S2 mbio.00679-23-s0004.xlsx The mutational backgrounds of the eight clusters of Figure 5.

Table S3 mbio.00679-23-s0005.xlsx Recurrent mutations with mutation times larger than 100.

Table S4 mbio.00679-23-s0006.xlsx Counts and proportions of three iSNVs categories for the groups of random and advantageous iSNVs.

Table S5 mbio.00679-23-s0007.xlsx Potential immune escape amino acid mutations in the RBD and NTD domains of the S protein.

ASM does not own the copyrights to Supplemental Material that may be linked to, or accessed through, an article. The authors have granted ASM a non-exclusive, world-wide license to publish the Supplemental Material files. Please contact the corresponding author directly for reuse.
