# Supplementary figures and images for: SARS-CoV-2 within-host diversity of human hosts and its implications for viral immune evasion
Source: mBio. 2023 Jun 5;14(4):e00679-23. doi: 10.1128/mbio.00679-23 (PMC10470530; doi:10.1128/mbio.00679-23)

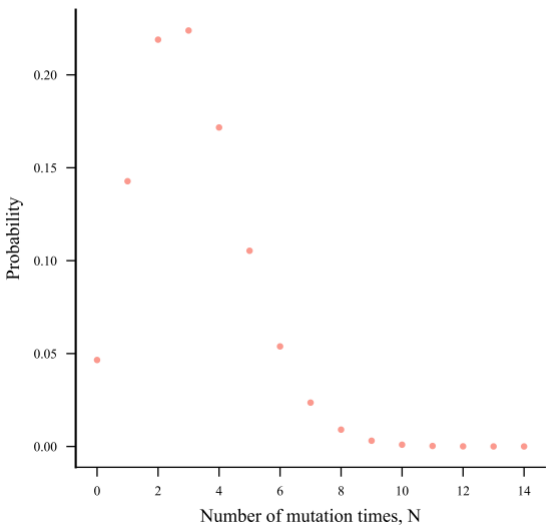

Supplement: Figure S1 — The probability distribution of mutation times at a particular genome position. Mutation times of a particular genome position in the dataset were modeled as a Poisson distribution with λ equal to 3.067, assuming that mutations occur randomly across the SARS-CoV-2 genome. [file mbio.00679-23-s0001.pdf]

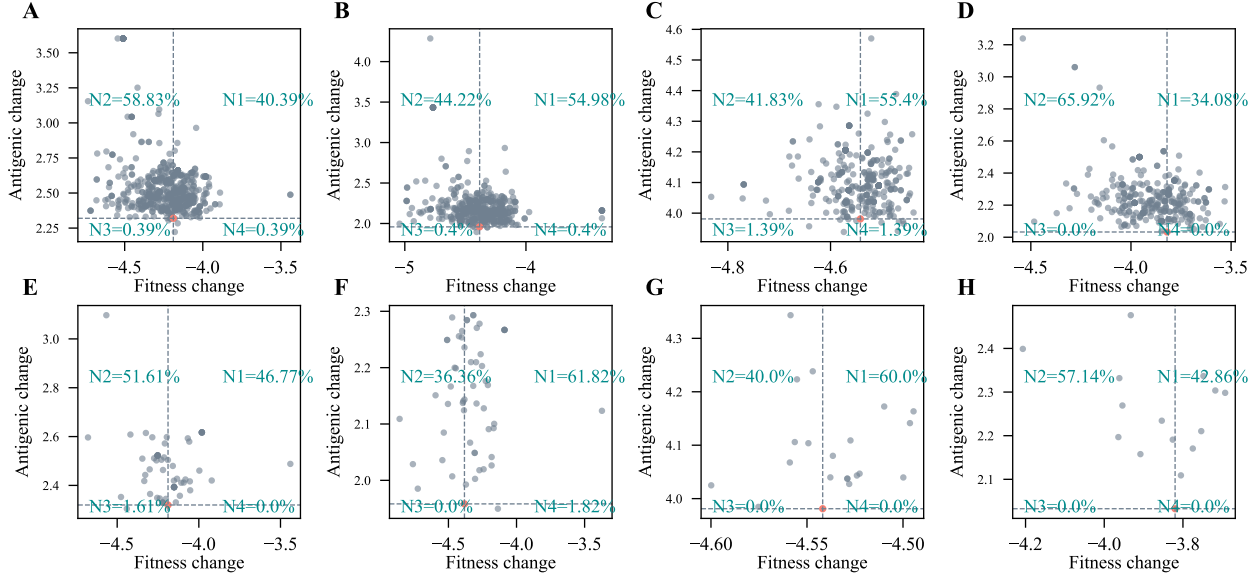

Supplement: Figure S2 — Prediction of immune escape ability of the random and advantageous iSNVs in the S gene. (A–D) The predicted results of the random iSNVs (iSNVs with AAF between 0.05 and 0.10). (E–H) The predicted results of the advantageous iSNVs (iSNVs with AAF between 0.90 and 0.95). (A) and (E), (B) and (F), (C) and (G), and (D) and (H) had the same mutational background, respectively. Furthermore, (A), (B), (C), and (D) had the same mutational backgrounds as Figure 5A, B, C, and D, respectively. The remaining four clusters in Figure 5 (Figure 5E through H) were not considered because of too few iSNVs with AAF between 0.90 and 0.95 in these clusters. [file mbio.00679-23-s0002.pdf]
